# Marine-Derived Biologically Active Compounds for the Potential Treatment of Rheumatoid Arthritis

**DOI:** 10.3390/md19010010

**Published:** 2020-12-29

**Authors:** Muhammad Bilal, Maimoona Qindeel, Leonardo Vieira Nunes, Marco Thúlio Saviatto Duarte, Luiz Fernando Romanholo Ferreira, Renato Nery Soriano, Hafiz M. N. Iqbal

**Affiliations:** 1 School of Life Science and Food Engineering, Huaiyin Institute of Technology, Huaian 223003, China; 2 Department of Pharmacy, Quaid-i-Azam University, Islamabad 45320, Pakistan; mqindeel@bs.qau.edu.pk; 3 Department of Medicine, Federal University of Juiz de Fora, Juiz de Fora-MG 36036-900, Brazil; leonardo.nunes@estudante.ufjf.br; 4 Department of Medicine, Federal University of Juiz de Fora, Governador Valadares-MG 35010-180, Brazil; marco.saviatto@estudante.ufjf.br; 5 Graduate Program in Process Engineering, Tiradentes University (UNIT), Av. Murilo Dantas, 300, Farolândia, Aracaju-Sergipe 49032-490, Brazil; luiz.fernando@souunit.com.br; 6 Institute of Technology and Research (ITP), Tiradentes University (UNIT), Av. Murilo Dantas, 300, Farolândia, Aracaju-Sergipe 49032-490, Brazil; 7 Division of Physiology and Biophysics, Department of Basic Life Sciences, Federal University of Juiz de Fora, Governador Valadares-MG 35010-180, Brazil; renato.soriano@ufjf.edu.br; 8 School of Engineering and Sciences, Tecnologico de Monterrey, Monterrey 64849, Mexico

**Keywords:** rheumatoid arthritis, marine-derived compounds, anti-inflammatory, drug development, biomedical applications

## Abstract

Rheumatoid arthritis (RA) is a chronic, systemic autoimmune disease with a prevalence rate of up to 1% and is significantly considered a common worldwide public health concern. Commercially, several traditional formulations are available to treat RA to some extent. However, these synthetic compounds exert toxicity and considerable side effects even at lower therapeutic concentrations. Considering the above-mentioned critiques, research is underway around the world in finding and exploiting potential alternatives. For instance, marine-derived biologically active compounds have gained much interest and are thus being extensively utilized to confront the confines of in practice counterparts, which have become ineffective for 21st-century medical settings. The utilization of naturally available bioactive compounds and their derivatives can minimize these synthetic compounds’ problems to treat RA. Several marine-derived compounds exhibit anti-inflammatory and antioxidant properties and can be effectively used for therapeutic purposes against RA. The results of several studies ensured that the extraction of biologically active compounds from marine sources could provide a new and safe source for drug development against RA. Finally, current challenges, gaps, and future perspectives have been included in this review.

## 1. Introduction

Rheumatoid arthritis (RA) is a chronic autoimmune disorder that affects the synovial joints lining and is allied with progressive disability, mortality, and socioeconomic burdens. The disease is more common in women than men, with a prevalence rate of 1%. The clinical sign and symptoms of RA include swelling, arthralgia, the loss of mobility, and redness of the joints [1,2]. The early diagnosis of RA is considered as the critical parameter to prevent disease progression. In the past 20 years, a significant advancement in the diagnosis and management of the disease has occurred. However, the early diagnosis remains challenging because it depends on the clinical information collected from the patient’s clinical and physical examination, including blood test and imaging results [3,4]. The pathogenesis of RA is allied with genetic factors and stimulated by environmental factors. It is proposed that almost 60–65% of RA cases are due to genetic factors. There are nearly 100 loci that are responsible for the progression of RA [5,6]. Among the several loci, the most common loci associated with RA contains information for the expression of major histocompatibility complex (MHC class-II), especially HLADRO1/O4. Additionally, the loci that express the post-translational modified enzyme, intercellular regulatory pathways, and costimulatory pathways also contribute towards disease progression [7]. The environmental factors like exposure to Epstein-barr virus, silica particles, alcohol, and obesity also possesses the capability to stimulate such genetic loci. The interaction among these genetic and environmental triggers leads to the onset of self-citrullinated proteins, which form autoantibodies against citrullinated peptides. The exposure of the lungs to several exogenous compounds like silica particles, Epstein-barr virus, *Aggregatibacter actinomycetemcomitans*, and *Porphyromonas gingivalis* can trigger the maturation of anti-citrullinated protein-antibody (ACPA) and citrullination of self-proteins [8]. This citrullination is catalyzed via the calcium-dependent enzyme, i.e., peptidylamine deaminase (PAD4), which converts the cationic arginine into neutral but polar citrulline. In RA patients, the PAD4 is secreted from the activated macrophages and granulocytes [9]. On the other hand, the level of ACPA is increased in RA patients due to an abnormal response of the immune system against various citrullinated proteins, including fibronectin, histones, type-II collagen, vimentin, and fibrin. These compounds also activate the MHC-class II, which subsequently stimulates the B-cells to produce further ACPA. This phase is also considered as the loss of tolerance. An interesting thing about the ACPA is that the level of these antibodies is detected years before the onset of RA symptoms, hence early detection and prophylactic treatment of these antibodies can reduce the severity and progression of the disease.

**Figure 1:**
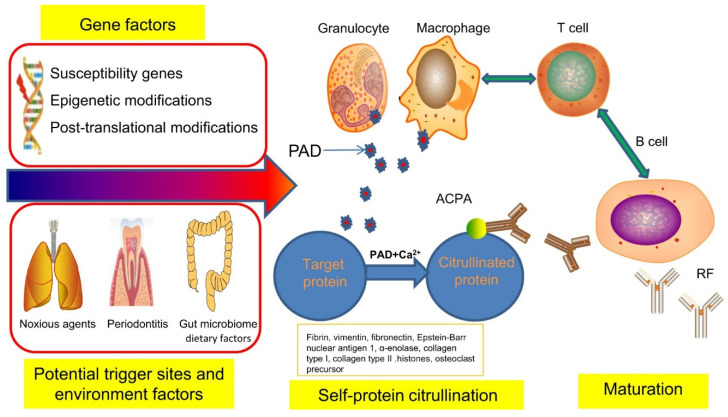
The visual illustration of the impact of several genetic and environmental factors in the pathogenesis of rheumatoid arthritis (RA). RF - rheumatoid factor. Reprinted from [10] with permission under a Creative Commons Attribution 4.0 International License. To view a copy of this license, visit http://creativecommons.org/licenses/by/4.0/. Copyright © The Author(s) 2018.

The visual illustration of the several stages involved in the development of RA is depicted in Figure 1 [10].

**Figure 2:**
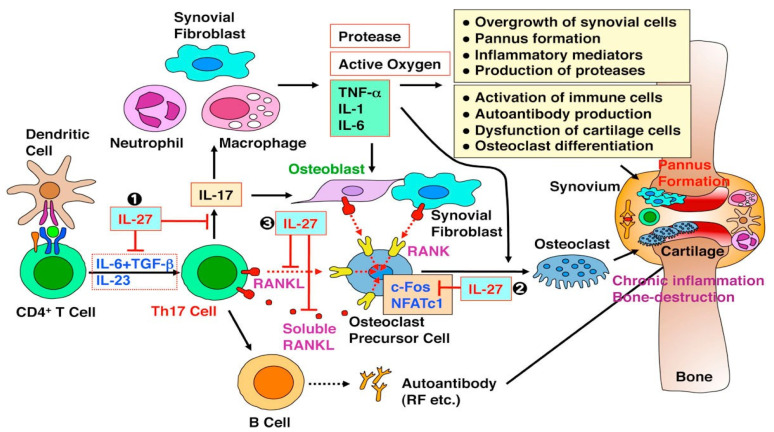
The schematic illustration of several mechanisms involved in the pathophysiology of RA. Reprinted from [17] with permission from under a Creative Commons Attribution 4.0 International License. To view a copy of this license, visit http://creativecommons.org/licenses/by/4.0/. Copyright © The Author(s) 2012.

RA’s etiology is linked with the imbalance of the immune system, and mostly, it develops in several phases [11]. In the starting phase, which is also known as a Pre-RA phase, the release of several inflammatory mediators and cytokines proceeds RA’s development. The significant changes that occur during this phase include changes in B- and T-cells regulation, the significantly higher formation of autoantibodies (that illustrate more specificity and affinity for modified proteins having citrulline residues), and alterations in the reactivity of the autoimmune system [12]. In the pre-RA phase, the autoantibodies’ affinity for carbamylated and acetylated peptides have also been found. The pre-RA phase consequently transforms into a clinically established RA phase, in which chronic inflammation and tissue remodeling have been observed [13]. RA’s pathophysiology is linked with the dysregulation of the immune system leading to high activation and penetration of macrophages, neutrophils, dendritic cells, and lymphocytes. These activation mechanisms subsequently lead to the formation of autoantibodies, which in turn possess the potential to detect several post-translational modified proteins [14]. The exact mechanism through which this autoreactivity transforms into chronic inflammation is not clear. The presence of these autoantibodies in the synovial fluids might happen due to localized microtrauma and complement activation [15]. Additionally, the circulating autoantibodies also detect the citrullinated proteins and immune complexes, which ultimately pledge the release of several inflammatory mediators (like IL-1β, IL-8, and IL-6, and tumor necrosis factor-alpha) and bone damage [16]. The visual illustration of the mechanism of alterations in the synovial membrane microenvironment is depicted in Figure 2 [17].

## 2. Literature Methodology—Inclusion/Exclusion Norms

Aiming to justify the current review theme and compilation, a standardized inclusion–exclusion criterion was implemented to scrutinize the collected literature from several authentic databases. Most of the earlier reported literature lacks such inclusion–exclusion norms to validate the literature search. For a said purpose, following two points were considered, i.e., (1) to conceptualize the scientific theme of the review, and (2) to critically cover and compile most recent and relevant literature contents. Two most authentic databases, i.e., Scopus and PubMed were searched by using the most relevant key terms. Upon literature collection, the data was carefully analyzed following the inclusion–exclusion criterion, i.e., the closely matched studies, as per the review theme, were included for further discussion and rest of the irrelevant or generalized studies were excluded without any consideration. More specifically, the pre-evaluation was performed considering the presence of all/any of the following keywords in the article title, abstract, and keywords, i.e., (1) conventional therapies against RA, and (2) marine compounds for RA. The data obtained from the Scopus database is summarized in Table 1. At Scopus, the literature search queries were performed on November 17, 2020, at https://www.scopus.com. While, in PubMed, the literature was searched for all previous years with the best match term on. Table 2 summarizes the search results obtained from the PubMed database. At PubMed, the literature search queries were performed on November 17, 2020, at https://pubmed.ncbi.nlm.nih.gov/. Based on the literature data obtained, the following sections and subsections were conceptualized and discussed with suitable examples as a core of this review.

## 3. Current Conventional Therapies against RA and Associated Problems

The pharmacological treatment of RA has advanced a lot in the last decades, allowing many patients to reach the state of remission or low disease activity, consequently improving the quality of life and limiting RA’s late complications. Early diagnosis and treatment are central to control inflammation and limit the damage. The continued treatment is equally important. However, a significant problem of conventional treatments of RA is the high costs, mainly disease modifying anti-rheumatic drugs (DMARDs) and targeted synthetic DMARDs. Therefore, the cost of treatment should be a factor considered by doctors in choosing the treatment [18]. In this context, studies about the possibility of reducing therapy in patients who have achieved remission status or low disease activity have been increasing, as it would be a desirable alternative for patients, who would be able to reduce their expenses with treatment. However, many concerns emerge as the possibility of increased cardiovascular risk and the occurrence of disease flare-up [19,20]. Another current discussion is about biosimilars, discovery and approval of generic compounds similar to biological DMARDs have grown, and some studies have shown equivalence of efficacy to the originals. Therefore, they constitute an essential alternative to reduce costs, offer more treatment options, and reduce inequalities in access to treatment between poor and rich countries [21]. Furthermore, as pointed Smolen et al. [22], therapeutic failure is an ordinary reality in RA patients. Several patients do not attain remission or low disease activity even if the treatment possibilities are exhausted, therefore, it is still necessary to discover new treatments and elucidate the mechanisms related to therapeutics failure and toxicity. Finally, one of the treatment’s biggest problems is related to the extensive side effects and complications, especially in patients with concomitant comorbidities. In addition to the high cost, the appearance of adverse consequences reduces the patient adherence to the medications [18]. The NSAIDs are adjuvant drugs in the symptomatic treatment of RA, promote rapid analgesia and reduce inflammation, can be prescribed, but are used on self-medication by most patients before seeking an expert and receiving the diagnosis. Prolonged use should be avoided because a significant number of side effects are related, some lighter as nausea and abdominal pain and others more severe as liver damage, bleeding by changes in coagulation and influence on renal circulation with nephrotoxic potential [23,24], increased risk of cardiovascular events, effects on blood pressure [25], and gastrointestinal related problems, such as ulcers and blending [26]. These adverse effects may vary according to the type of nonsteroidal anti-inflammatory drugs (NSAIDs) and some can be controlled with other drugs such as antacids and proton pump inhibitors or changes in diet [27]. Thus, attention is needed when prescribing NSAIDs in the treatment of RA and contraindications, especially in groups with a greater chance of adverse events such as patients with renal or hepatic dysfunction, systemic arterial hypertension, intestinal diseases, and blood clotting disorders [23].

Glucocorticoids are more potent than NSAIDs and are often prescribed in combination with DMARDs and cases of severe systemic manifestations of RA. According to Strehl et al. [28], many adverse effects can occur. The extent of them seems to be associated with the specific conditions of the treatment (dosage and duration) and the particular patient. Among them are reported in the literature, mainly altered bone metabolism and increased risk of fractures [29], weight gain, increased risk of infections, changes in hormone secretion [30], insulin resistance, and diabetes [31]. Additionally, there seems to be a combination of them with a risk increase of cardiovascular events; however, with insufficient evidence in the literature [32]. Some care is essential during the use of glucocorticoids, careful monitoring of patients should be undertaken and preventive measures such as low doses and time of limited use implemented [33], and it special attention is required for patients with comorbidities that can be aggravated by these adverse effects such as diabetes, hypertension, and dyslipidemia [28]. There are several DMARDs available for the treatment of RA, each of them has specific complications and problems. Still, in general, they have an excellent risk–benefit given the modifying effect in the disease course. Among conventional DMARDs, methotrexate (MTX), (i.e., first-line agent against RA) is related to an increased risk of adverse pulmonary events [34], alteration of hepatic transaminases [35], bone marrow deterioration, and, in rare cases, neurological symptoms. The supplementation of folic acid is a highly recommended pair to reduce hepatic adverse effects [36]. Leflunomide is associated with flares, gastrointestinal events, allergic reactions, infections, and hypertension [37]. The biological DMARDs also have an excellent benefit–risk profile, however, have an increased risk of serious infections compared to conventional DMARDs, it is important that the rate of infections can vary depending on other underlying risk factors [38]. Considering the different properties and mechanisms of biological ones, there is a risk of reactivation of tuberculosis, mainly for TNF inhibitors [39], which were also related to the worsening of multiple sclerosis crises [40]. The literature also reports a risk of intestinal perforation in patients treated with tocilizumab [41]. Regarding targeted synthetic DMARDs, data are still limited in the literature since they are newer drugs, and there are many clinical trials still underway. In general, JAK inhibitors have adverse effects similar to biological DMARDs [42]. Still, there is an increased risk of herpes zoster infection [43,44], venous thromboembolism has been associated with the tofacitinib and baricitinib, especially in patients with risk profile for these events and older [18].

**Figure 3:**
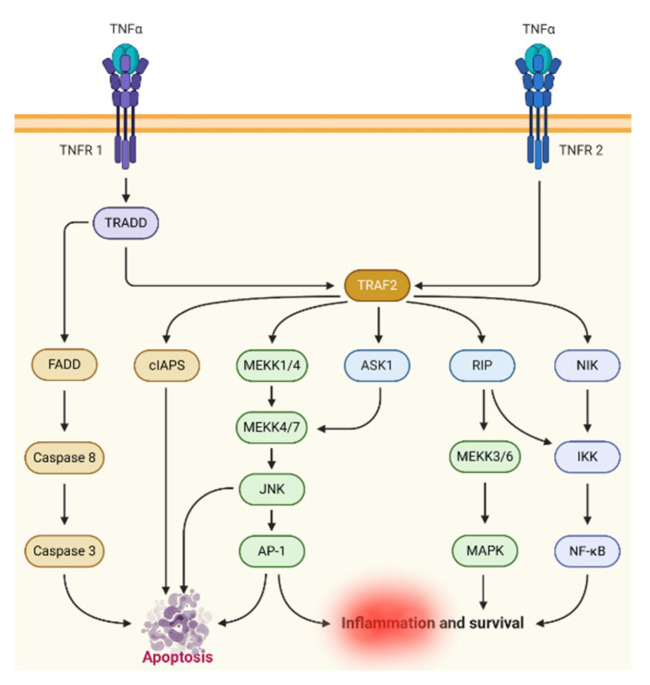
TNF-alpha. The figure was created with the “BioRender.com” template and exported under the terms of premium subscription.

TNF-alpha is produced from the activated macrophages, monocytes, and T-lymphocytes, and triggers the inflammatory responses (Figure 3). The higher expression of TNF-alpha mediates the destruction of the bones and ultimately stimulates the progression of the disease. Therefore, various TNF-alpha inhibitors were introduced as agents for therapy against RA [45]. Infliximab was the first chimeric monoclonal antibody having mouse idiotype and human antibody backbone. This antibody possesses the potential to counteract the biological activity of TNF-alpha by binding with all forms of the TNF-alpha [46]. It is administered as an intravenous infusion and exhibits a long-term safety profile. The patients treated with infliximab demonstrate a significant reduction of adhesion molecules, including IL-8, IL-6, MCP-1, and IL-1. Despite the safety profile, the infliximab exhibits severe adverse effects such as reactivation of tuberculosis or hepatitis B, cancers, and lymphoma [47]. Adalimumab is another example of a TNF-alpha inhibitor, a wholly humanized antibody, and administered through a subcutaneous route. It exhibits less toxicity profile and produces effects when used in combination with MTX. The common adverse effects include latent reactions, skin reactions, and cardiac arrest [48]. Etanercept is a hybrid protein consisting of human TNF receptors and immunoglobulin backbone. It is also administered through the subcutaneous route twice a week. It also exhibits the same toxicity profile as that of adalimumab and infliximab. Golimumab is another example used to inhibit TNF-alpha and is administered once a month through a subcutaneous route. The significant adverse effects include cancers, tuberculosis, and severe infections [49]. Additionally, all the TNF-alpha inhibitors show the loss of response with the passage of time and hence the patients have to switch to other biologics.

Most T-cells penetrate in the synovium and some of them penetrate in the synovial fluids and increase the expression of proinflammatory cytokines, like TNF-alpha and interferon, which leads to cartilage damage, bone erosion, and pannus tissue formation. To counteract this mechanism, various T-cell targeted therapies have been devised. One such example includes the abatacept, a T-cell costimulation modulator consisting of an extracellular domain connected with the modified Fc fragment of the IgG1 [50]. The abatacept interacts and inhibits the signaling between CD 80 and CD 86 and, in this way, exhibits its efficacy. It is available in the form of injection and infusion. The most common side effects include sore throat, headache, common cold, infection, and nausea [51]. In RA, the IL-6 stimulates pannus formation via increased expression of vascular endothelial growth factor and ultimately increases the bone resorption. Tocilizumab is a human-based antibody that specifically targets the IL-6. It is available as an intravenous and subcutaneous formulation and exhibits less immunogenicity [52]. The other examples include sirukumab, clazakizumab, olokizumab, and sariliumab. The common adverse effects of these therapies include hypertension, headaches, and respiratory tract infections. Further clinical trials are also required to validate further these agents’ therapeutic efficacy against RA [53].

IL-1 is a proinflammatory cytokine that possesses the potential to produce proinflammatory actions. Some treatments are also available for targeted therapy against this interleukin [54]. Anakinra is one such example that acts as an IL-1 receptor antagonist and is administered as a once-daily injection. It blocks the activity of IL-1alpha and IL-1beta by explicitly blocking the IL-1 receptors. The major drawbacks of these formulations include itchy rashes, upper respiratory tract infections, allergy, and gastrointestinal tract infections [55]. B-lymphocytes produce their inflammatory properties owing to their activity on the antigen presentation and through the expression of proinflammatory cytokines. Rituximab is an antibody that specifically targets the CD-20 positive B-lymphocytes. The binding of the antibody with CD-20 allows the rituximab to decrease B-lymphocytes’ functional responses through complement-dependent cytotoxicity, cell mediation, and promotion of growth arrest and apoptosis [56]. Belimumab is another example of this class that binds with the B-lymphocyte stimulator antibody (BLYS). The level of BLYS is significantly increased in the serum and synovial fluid of RA positive patients. This BLYS is very important for B-cells’ persistence and its blockage can cause apoptosis of the autoimmune B-cells [57].

## 4. Marine-Derived Biologically Active Compounds

The oceans are home to many biological and chemical compounds and have enormous biodiversity globally, with about 80% of the world’s animal and plant species [58,59]. The oceanic environment is hostile and competitive, conditioning marine organisms to develop adaptive mechanisms through biochemical compounds to resist various types of stressors. Thus, the metabolites produced give these organisms unique structural and functional characteristics [60,61]. As Halvey [62] points out, life originated in the sea and adapted to the terrestrial environment throughout evolution. Despite intense structural changes, many molecules continue to have the same physiological functions. Therefore, several bioactive compounds of marine organisms have therapeutic potential and may be candidates for developing drugs and products for the treatment of human diseases [63].

Discoveries and studies of marine bioactive compounds are still recent compared to other areas of knowledge. In recent decades, numerous new molecules have been documented, patented, and already tested in clinical trials [61,64]. Approximately 25,000 marine chemical compounds have been reported [65]. With the improvement of the technologies of exploitation and extraction of these compounds and the undeniable therapeutic potential, the trend is that in the coming years many drugs, supplements, and natural products with marine derivatives will emerge to treat a multitude of diseases [63]. Several studies have shown that bioactive marine compounds have significant antitumor and anticancer activities [66]. Jimenez et al. [67] analyzed five marine-derived drugs successfully against cancer, and several other diseases in clinical trials. The literature also reports the association of these compounds with several other therapeutic effects such as treatment of diabetes, chronic pain, and cardiovascular diseases, and antibacterial, antifungal, antiprotozoal, antituberculosis, and antiviral activity [68]. Finally, marine-derived biologically active compounds can be used in immunotherapies. They can act by inducing, increasing, or reducing the immune response, therefore, with enormous potential for therapeutic use [60]. In this context, evidence and findings have pointed out several marine derivatives with immunomodulatory and anti-inflammatory properties [60,69,70,71], which represents new sources for the treatment, damage control, and prevention of rheumatologic diseases whose etiopathogenesis involves inflammatory pathway disorders, such as RA.

### 4.1. Glycosaminoglycans—Chondroitin Sulfate and Hyaluronic Acid

Glycosaminoglycan (GAGs) are multifunctional polysaccharides composed of repeating disaccharide units that may change the form of sulfation and epimerization, which determines different functions of protein recognition and biological activities of these compounds [72,73]. Two important groups of complex heteropolysaccharides belonging to the class of GAGs are chondroitin sulfate (CS) and hyaluronic acid (HA). CS is formed by repeated disaccharides *N*-acetyl-d-galactosamine (GalNAc) and d-glucuronic acid (GlcA) with sulfate groups allocated in different numbers and positions, which makes this polymer extremely heterogeneous in terms of length and structure [74,75]. Around 16 various disaccharides can be formed depending on the position of sulfation [76], and there are differences in concentration and composition between land and marine source SC. It is a biomolecule present in virtually all vertebrate organisms and invertebrates, mostly marine organisms, because they present unusual sulfation patterns. Consequently, it is involved in many biological processes at the molecular, cellular, and tissue levels [75,77,78]. They play an essential structural role in the composition of extracellular matrix and formation of tissues such as cartilage and bones, abundant in mammals’ connective tissue [73,79]. Some studies have revealed that CS has immunomodulatory and anti-inflammatory properties in several diseases [80,81], highlighting the promising effects of CS reducing symptoms and improving function in osteoarthritis patients, which is one of the consequences of RA in advanced phases [82,83]. According to Abdallah et al. [72] compiled in a recent review, CS can be extracted from cartilage, head, skeleton, fins, and skin from different marine animals such as sharks, salmon, zebrafish, and other species of fish, squid, ray, and octopus. Still, the primary marine source in commercial terms is shark cartilage. Therefore, they are valuable compounds that can be collected to optimize the use of marine waste.

HA consists of units of disaccharides *N*-acetyl-d-glucosamine (GalNAc) and d-glucuronic acid (GlcA) [84], is an unbranched high molecular weight linear polysaccharide, the only nonsulfated GAGs that is not bound to proteins [85,86]. It is widely distributed in the conjunctive as an essential component of the extracellular matrix, playing a vital role in controlling tissue permeability and hydration, macromolecular transport between cells, and bacterial invasion control [85,87]. The human body is found in higher concentrations in connective tissues such as synovial fluid, the vitreous humor of the eyeball, and the umbilical cord [87]. HA from marine sources can be extracted mainly from the eyeball and liver of swordfish, shark, mollusk bivalves, stingray, and tuna [72,87]. HA is widely used in the biomedical sector for the production of hydrogels that can be used as long-term low-dose drug delivery vehicles [88,89], with an input for the development of new biomaterials applied to wound healing [90] and tissue culture scaffolds [88,91,92]. Evidence has revealed that HA has anti-inflammatory properties and has been used in RA treatment for decades [89,91,93].

### 4.2. Chitin and Chitosan

Chitin or poly (β-(1→4)-*N*-acetyl-d-glucosamine) is a polysaccharide synthesized by numerous living organisms and one of the most abundant natural biopolymers on earth. It is found in the exoskeleton of crustaceans and cell walls of marine fungi [94], but is extracted mainly from the shell of the crab, shrimp, and lobster [95]. Due to its characteristics of high strength, biocompatibility, high biodegradability, and low toxicity, it is a biopolymer with numerous applications in the biomedical field, for example, gene delivery, target drug delivery, surgical sutures, and tissue engineering products [96,97,98].

Chitosan is the direct derivative of chitin obtained by partial deacetylation under alkaline conditions [95], shares characteristics similar to its precursor. Still, chitosan has more applications in the chemical areas, nutraceutical, and pharmaceutical industries [73,99]. It has hydrophilic and antimicrobial properties, being necessary for the production of biomaterials [95]. It is interesting for application in drug delivery systems, emphasizing the development of chitosan-based nanosystems to treat inflammatory diseases such as RA [100]. Studies have pointed out that chitosan exerts anti-inflammatory, antioxidant, antimicrobial, antitumor, and hypocholesterolemic activity [101,102,103,104].

### 4.3. Alginate—Polysaccharides

Alginate is a natural polysaccharide composed of building blocks of 1,4-linked (-d-mannuronic acid) (M) and (-l-guluronic acid) (G), which vary in proportion forming alginate compounds with different chemical and physical characteristics [105,106]. The primary sources of Alginate are brown seaweed such as *Ascophyllum nodosum, Laminaria hyperborea, Saccharina japonica, Macrocystis pyrifera,* and *Laminaria digitata* [107]. It is bioactive with biocompatibility, low cost, low toxicity, gelling agent, and stabilizer of solutions, which make it interesting for various biomedical applications [108], nutraceuticals, and cosmetics [109]. It is used to treat wounds, and there are already at least 12 commercially available alginate-based dressings with promising results due to its immunogenic, antibacterial, and procoagulant activities [107,110].

### 4.4. Peptides

Peptides play numerous bioregulatory roles of extreme importance. Those of marine origin stand out for having unique molecular mechanisms [111]. They offer enormous possibilities for the study of several secondary metabolites, which have high specificity and low toxicity. Therefore, they constitute an opportunity to identify new prototypes of drugs and products, expanding their applications in the pharmaceutical and biomedical industry [111,112]. Bioactive peptides usually have 3–20 amino acid residues organized in different sequences, determining distinct structures and properties [113]. Given the various possible compositions, they can perform different biological activities such as antiviral, antifungal, anticancer, antidiabetic, antioxidant, anticoagulant, antihypertensive, immunomodulatory, analgesic, and calcium-binding properties, and most marine peptides have antimicrobial activity [114,115]. The extraction of marine bioactive peptides is made from bacteria, mainly marine cyanobacteria, microalgae such as *Chlorella vulgaris* (green algae), marine sponges, and their associated microorganisms [114,116].

### 4.5. Fatty Acids

**Figure 4:**
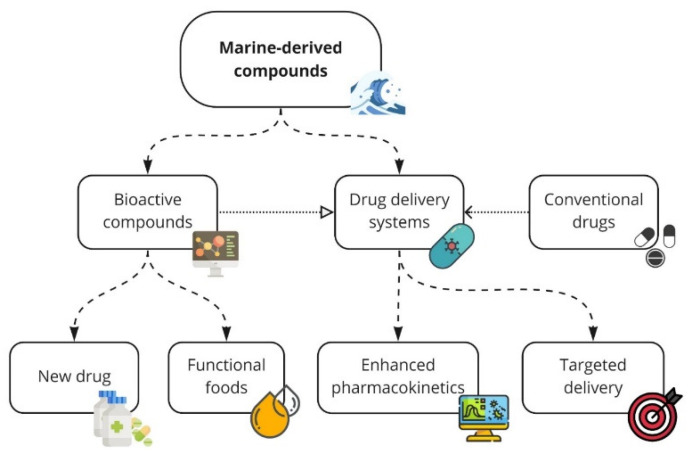
Strategies for implementing marine-derived compounds for RA treatment. Marine-derived compounds can form new bioactive substances and new drug delivery systems. These bioactive compounds can be implemented for RA treatment as new drugs or functional foods. The drug delivery systems can be applied to enhance the pharmacokinetic properties of both marine-derived bioactive compounds and conventional drugs and deliver these substances on-target.

Fatty acids (FA) are carboxylic acids with different carbon numbers and double bonds. According to the structure and biochemical properties they are classified into two broad groups, saturated FAs that do not contain double bonds in their carbon structure and unsaturated FAs that include double bonds in their composition and are subdivided into monounsaturated FAs (MUFAs) or polyunsaturated FAs (PUFAs) [117,118]. PUFAs are classified into two categories: (i) Omega-3 (n-3 PUFAs), which mainly includes α-Linolenic acid (ALA), eicosapentaenoic acid (EPA), and docosahexaenoic acid (DHA); (ii) Omega-6 (n-6 PUFAs), which includes linoleic acid (LA); y-linoleic acid (GLA) and arachidonic acid (AA) [119,120]. They are synthesized by the human organism but need to be also ingested through diet, being classified as essential FAs due to their enormous importance participating in various metabolic processes throughout human life [120] and constitute the phospholipids that form the cell membrane [121]. They act significantly in inflammatory responses with participation as substrates for the biosynthesis of inflammatory mediators, cellular receptors’ activation, and modulation of membrane fluidity to alter cell function [122,123,124]. Omega-3 rich oils, especially DHA and EPA, can be extracted from seafood such as algae and fatty fish, the best are salmon, sardines, tuna, herring, and trout [119,125]. Marine fatty acids play essential anti-inflammatory activities and studies have pointed out that they can be used in the treatment of RA, promoting clinical improvements [126,127,128,129]. Figure 4 is showing the various strategies that can be implemented to treat RA effectively.

## 5. Advantages and Applications of Various Marine-Derived Compounds for RA Therapy

Over the past two decades, a better understanding of the pathophysiology of RA has allowed significant progress in the treatment efficacy. Multiple possibilities of intervention arose from comprehending the complex pathways involved in the inflammation [130]. Despite all the advances, still 20–25% of the patients cannot reach low disease activity with all options available [131]. On the other hand, this noticeable room for improvement could be fulfilled by the inexhaustible source of unique and useful compounds: the marine environment. Many strategies can be applied in the process of employing these products in the context of RA. Its benefits could range from improving the effectiveness of already known drugs, diminishing the adverse effects and lowering the costs of treatment, to the discovery of new medicines that could act via the established mechanisms as well as through others not yet explored in this particular disease.

Methotrexate is an excellent example of a drug that could have its pharmacokinetics properties improved. It has been used in RA for more than 50 years. It is still a part of the first-line approach to the disease [131], even though its rapid elimination by the kidney grants it a relatively short half-life on plasma, resulting in low drug concentration in the target tissue [132], a characteristic far from ideal, considering the long-term therapy needed in RA. This rapid excretion is an important aspect, as it has to be compensated by more frequent and higher doses to maintain the desired effect [133], which comes with an increased risk of therapeutic tolerance and systemic adverse effects [134]. Under these circumstances, drug delivery systems, such as liposomes, nanoparticles, and microspheres, would help increase solubility, bioavailability, half-life, and drug action on the inflammation sites while minimizing systemic exposure and adverse effects [135,136,137,138,139]. By improving the impact of the first-line treatment approach, it would be possible to avoid the employment of biologic DMARDs, whose price may reach five-digits [131], hence reducing the overall cost of the therapy.

**Figure 5:**
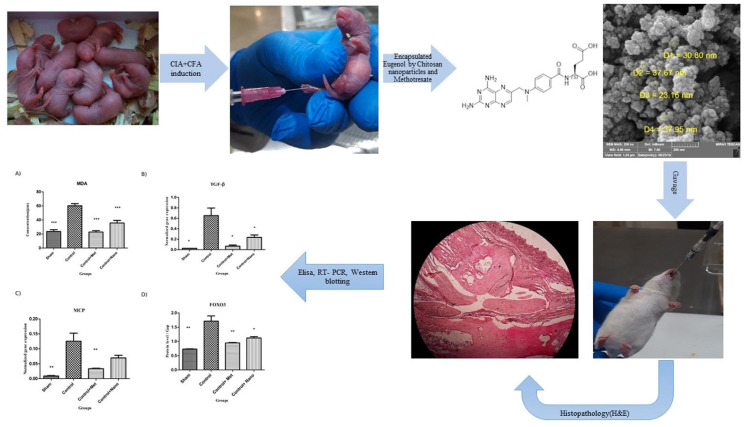
Evaluation of Encapsulated Eugenol by Chitosan Nanoparticles on the aggressive model of rheumatoid arthritis. Reprinted from [148] with permission from Elsevier. © 2020 Elsevier B.V.

For a compound to be used in a drug delivery system, it must present biocompatibility, low immunogenicity, and low toxicity. Furthermore, it is also relevant that it can be modified [132,140,141]. These properties are widely observed in marine-derived natural polymers such as carrageenan, fucoidans, alginate, and agar, making them potential bases for producing these systems [142]. Indeed, there has been extensive research using these compounds for delivering drugs for treating diabetes, pain, infections, and cancer, some with the ability to target specific cells and some capable of producing particles with size ranging from 1 nm up to 1000 nm [143]. When it comes to RA therapy, many researchers were able to employ these exciting properties of the marine-derived compounds for modifying pharmacokinetics of other drugs to control inflammation [144]. A platform of alginate beads has successfully delayed the release of Prednisolone in rats [145]. A chitosan thermosensitive hydrogel combined with alginate microspheres could prolong the release of Diclofenac sodium to 5 days in vitro and present promising characteristics for intra-articular administrations [146]. Sodium alginate has been used to prepare Ibuprofen microbeads with a variety of physicochemical properties [147]. Encapsulated Eugenol with Chitosan Nanoparticle has been able to alleviate the symptoms of joint inflammation, synovial hyperplasia, and cartilage damage caused by RA in rats (Figure 5) [148]. Chitosan improved Leflunomide’s anti-arthritic effect when used as a coating in an oral nanosystem in an RA-induced rat model. Chondroitin sulfate was also used in this same investigation as a coating and showed even better joint healing, probably due to its cartilage homing process [149]. A platelet-rich plasma (PRP)-chitosan thermo-responsive hydrogel was able to reduce edema degree on an arthritic rat model when combined with black phosphorus nanosheets (BPN) [150]. Carboxymethyl chitosan has been proven to be a good carrier for the treatment of RA, as it was able to enhance Triptolide’s solubility and reduced its toxicity both in vitro and in vivo [151]. Chitosan nanoparticles were also used to encapsulate Methotrexate and Dexamethasone and showed promising results in controlling inflammation in a rat arthritic model after intraperitoneal administration [152]. Other Methotrexate conjugated nanoparticles were engineered based on chitosan and demonstrated the potential for treating ovarian cancer [140]. Another complex sialic acid (SA)-modified chitosan oligosaccharide-based biphasic calcium phosphate (BCP) loaded with Methotrexate not only was capable of a targeted delivery into arthritic paws but executed a rapid drug release and significantly inhibited the inflammation response. The component also enhanced bone regeneration, expanding the treatment of RA for a nanometer-scale dimension, and acting further than only aiming at low disease activity or remission [153], as even the most effective conventional therapy will not reverse the joint damage [131].

As if the benefits and possibilities of modifying already known drugs were not enough, the lengthy list of bioactive marine substances that shows analgesic, antitumor, immunomodulatory, antioxidant, and anti-inflammatory properties makes the oceans a relevant source of new therapies for RA [60,69,141,142,154]. The anti-inflammatory mechanisms reported are very diverse and could allow a more complex approach to the pathways responsible for RA’s development. In other words, these marine substances could represent multiple possibilities to interfere in different steps of the pathogenesis with varying intensities from the ones now applied, restoring the balance between the pro and anti-inflammatory cytokines [155]. If correctly employed, this variety of options can certainly make it possible to explore the alluring spectrum of precision medicine to provide a more adequate treatment for each individual [156] in a therapy that better considers factors like the disease level of activity, the presence of comorbid conditions, the stage of therapy, the patient preferences, and the presence of adverse prognostic signs for each specific case [157]. Besides, this precision treatment based on the patient profile could be even more helpful for those who could not reach low disease activity or remission with the currently offered treatments [156].

**Figure 6:**
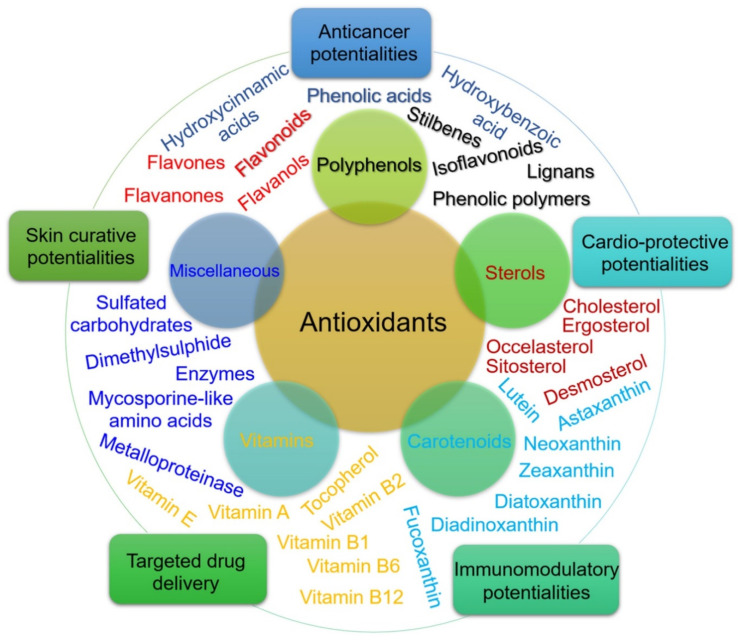
An array of algal-based antioxidants. The inner circle shows the diversity of biologically active antioxidants produced in the marine environment. The outer circle represents their novel therapeutic and biomedical potentialities. Reprinted from [158] with permission from Elsevier. © 2020 Elsevier B.V.

The application of marine compounds could take place as a complementary medicine implemented through diet and the development of a new drug. It has been pointed out that 30–60% of rheumatic patients use complementary medicine [158]. The marine world represents a vast reserve of anti-inflammatory and antioxidant substances (Figure 6) [158], that might be helpful against chronic inflammatory diseases like RA, such as carotenoids, n-3 polyunsaturated fatty acids (n-3 PUFAs), vitamins, and peptides [154]. A systematic review and meta-analysis of randomized trials found moderate-quality evidence for marine oil use to alleviate pain in RA [128]. In another study, the supplementation with n-3 PUFAs mostly obtained from fish has been considered a valuable option for RA, as it was able to reduce the expression of TNF-α and interleukin-1β, and improved the pain symptoms, the tender joint count, the duration of morning stiffness, and the frequency of NSAIDs consumption [156,159]. A list of recent patents and discoveries of marine anti-inflammatory agents named mussel lipids as applicable to RA [160].

Since antioxidants can also suppress the release of inflammatory cytokines, reducing reactive oxygen species (ROS) production, and scavenging free radicals systemically, marine carotenoids (e.g., Astaxanthin and Fucoxanthin), polysaccharides, and phenols have been assessed for their potential role as functional foods in RA [154,161]. It is stated that a higher intake of these components can not only alleviate symptoms but also ameliorate adverse effects and risk of complications of pharmacological therapy and prevent RA development [154]. However, it is important to highlight that the mechanisms of these components still have to be elucidated [155]. The evidence for the practical use of these substances is still scarce [162]. There is usually a lack of communication between doctors and patients using natural products for RA that can be prejudicial to the therapy [163].

Marine-derived compounds can also be explored in the discovery of new drugs that could act both on well-known pathways (with fewer adverse effects) and on others not yet seen as suitable for RA. For instance, 4-(Hydroxymethyl)catechol extracted from fungi in marine sponges was able to modulate the PI3K/Akt/NF-kB pathway, suppressing the Th immune responses and matrix metalloproteinases expression, hence inhibiting the production of inflammatory cytokines in human RA synovial fibroblasts which confirmed its potential anti-RA effect [164]. Steroids obtained from a bivalve (*Paphia malabarica*) showed important antioxidant and anti-inflammatory activities and could have an essential role in RA therapy [165]. A fucoidan like sulfated polysaccharide extracted from the macroalgae *Turbinaria ornata* inhibited inflammation and bone damage with a significant reduction in the arthritic score and paw volume in rats [166]. Recently, a few marine compounds have been described as ligands of the Pregnane X Receptor (PXR), a nuclear membrane receptor involved in crucial physiological processes as detoxification, glucose and lipid homeostasis, bone metabolism, and inflammation [167,168]. Solomonsterol A, an agonist of the PXR extracted from the marine sponge *Theonella swinhoei*, is capable of attenuating systemic inflammation and immune dysfunction in a mouse model of RA [169], which enforces the possibility of finding new pathways to intervene in the pathological process of RA. The details of various marine-derived compounds for the potential treatment of RA are illustrated in Table 3.

## 6. Concluding Remarks, Challenges, and Future Outlook

The current review focused on some marine-derived compounds for the potential treatment of RA. In addition to the valuable nutritional values, these natural compounds and their derivatives have demonstrated anti-inflammatory and antioxidant properties and thus could be used as drug candidates for therapy against RA. with an increment in the exploration of the marine sources, it is anticipated that more novel compounds with anti-inflammatory and analgesic properties will be explored and subsequently developed as antiarthritic agents for clinical use.

Despite the plethora of possibilities described, only a few marine compounds were explicitly tested for RA, and no new drug has yet been approved for this purpose. This contrasts with anticancer therapy, which had in 2017 seven marine-derived pharmaceutical substances authorized by the Food and Drugs Administration for clinical use as drugs. Others underwent different stages of clinical trials in oncology and hematology [172]. Indeed, this advance against tumors is good news for overall marine research and its applicability in RA, as the two diseases share a few mechanisms in their pathogenesis, such as the participation of the immune system and cytokines, intense cell proliferation, and angiogenesis [132]. Hence, the pharmacological compounds developed for cancer might as well be useful for the treatment and diagnosis of RA.

The road to developing a new drug is long and takes a lot of time, investment, and hard work. Besides, it becomes even more complicated with so many possibilities to be screened. On the other hand, considering the low overall success rate, it is essential to maintain a robust pipeline of new drug candidates in which marine natural products could make a significant contribution [173]. To overcome these challenges, innovative approaches such as artificial intelligence (AI) can be implemented to make the hunt for new medicines quicker, cheaper, and more effective [174]. Indeed, AI represents a powerful data mining tool that can be used in many phases of the drug developing process such as virtual screening, quantitative structure–activity relationship (QSAR) analysis, de novo drug design, activity scoring, and in silico assessment of absorption, distribution, metabolism, excretion, and toxicity (ADME/T) properties [175].

One relevant problem when using natural products is their low bioavailability. Still, the development of nanoparticles, even marine-derived ones, for delivering these drugs can solve it, protecting the substances against degradation and delivering them in specific tissues [141]. Lastly, another challenge is to escalate the production for a pharmaceutical application, as often, an insufficient quantity of a compound of interest can be isolated from marine organisms [69]. To surpass this, a pharmacophore analog that replicates the lead natural substance’s desired biological activity can be developed, ideally in a more straightforward, more potent, and less toxic way [173]. Another option that can be used for some organisms is to find new ways of increasing their cultivation. For instance, new aquaculture technologies have been made possible for various types of soft corals, helping to solve this issue [69].

## Figures and Tables

**Table 1:** Literature quest results attained from the Scopus database. The spreading of articles in each examination group is based on total number of articles and reads from top to bottom column wise.

Search Terms	Document Types	No. of Articles from All Years		# of Articles from Top Journals		# of Articles Based on Territory	
Conventional therapies against rheumatoid arthritis	Article, Review, Book Chapter, Conference Paper, Book	2020	20	Annals of the Rheumatic Diseases	15	United Kingdom	53
		2019	23	Clinical Rheumatology	06	United States	45
		2018	16	Advances in Therapy	04	Germany	32
		2017	20	Archives of Rheumatology	04	Italy	26
		2016	11	Clinical and Experimental Rheumatology	04	France	25
		All past years	135	All other journals	192	Rest of the countries	41
Marine compounds for rheumatoid arthritis	Article, Review, Book Chapter, Conference Paper	2020	03	Marine Drugs	06	India	05
		2019	01	Current Medicinal Chemistry	03	Ireland	03
		2018	03	Frontiers in Pharmacology	02	South Korea	03
		2017	03	PLOS ONE	02	Australia	02
		2016	02	Progress in Drug Research	02	Bangladesh	02
		All past years	17	All other journals	14	Rest of the countries	14

**Table 2:** Literature quest results attained from the PubMed database.

Search Terms	Total Articles	No. of Articles Published in the Last Five Years Filtered with Best Match Term on					
		2020	2019	2018	2017	2016	All Past Years
Conventional therapies against rheumatoid arthritis	237	20	27	21	20	14	135
Marine compounds for rheumatoid arthritis	18	01	01	03	03	00	10

**Table 3:** Applications of marine-derived compounds for therapy against RA.

Marine-Derived Compound	Source(s)	Applications for Therapy against RA	Reference
*n*-3 PUFAs	Fish oil	Reduce the expression of TNF-α and interleukin-1β, pain symptoms, the duration of morning stiffness, and the frequency of NSAIDs consumption Moderate-quality evidence for the use of marine oil to alleviate pain in RA	[128,156,159]
	Mussel (*Mytilus coruscus* and *Perna canaliculus*)	Can be used for the prevention and treatment of RA A synergistic effect is obtained of combined omega-3 series fatty acid and flavonoids in the treatment of RA	[160,170,171]
Astaxanthin	Algae and aquatic animals	Potential prevention and treatment of RA due to antioxidant and membrane preservation properties	[154]
Fucoxanthin	Marine brown seaweeds	Potential prevention and treatment of RA due to powerful antioxidant properties	[154]
4-(Hydroxymethyl)catechol	Fungi in marine sponges	Modulate the PI3K/Akt/NF-kB pathway, suppressing the Th immune responses and matrix metalloproteinases expression, thus inhibiting the production of inflammatory cytokines in human RA synovial fibroblasts	[164]
Steroids	Bivalve (*Paphia malabarica*)	Antioxidant and anti-inflammatory activities and may play an important role in RA therapy	[165]
Fucoidan	Macroalgae (*Turbinaria ornata)*	Inhibits inflammation and bone damage with a significant reduction in the arthritic score and paw volume in rats	[166]
Solomonsterol A	Marine sponge (*Theonella swinhoei)*	Attenuates systemic inflammation and immune dysfunction in a mouse model of RA	[169]

*n*-3 PUFAs: *n*-3 polyunsaturated fatty acids; RA: rheumatoid arthritis; NSAIDs: nonsteroidal anti-inflammatory drugs.
